# Correction to: ‘Assessing the stability of human locomotion: a review of current measures’ (2013) by Bruijn *et al.*

**DOI:** 10.1098/rsif.2023.0207

**Published:** 2023-05-03

**Authors:** S. M. Bruijn, O. G. Meijer, P. J. Beek, J. H. van Dieën

*J. R. Soc. Interface* **10**, 20120999. (Published online 6 June 2013) (https://doi.org/10.1098/rsif.2012.0999)

An attentive reader pointed out a mistake in our paper. On page 6 the paper reads ‘Chang *et al.* [95] reported that walking over an unstable support surface increases values of *λ_S_*, but not of *λ_L_*.’, which actually should have read:

‘Chang *et al.* [95] reported that walking over an unstable support surface decreases values of *λ_L_*, but not of *λ_S_*.’.

This mistake does not further influence the conclusions we reach regarding these stability measures.

